# OCT Angiography and En Face OCT Reflectance Aid in Monitoring Subclinical Inflammation in Serpiginous Choroidopathy

**DOI:** 10.1155/2020/8897394

**Published:** 2020-07-27

**Authors:** Carl S. Wilkins, Jorge S. Andrade Romo, Toco Y. P. Chui, Richard B. Rosen, Stephanie Llop

**Affiliations:** Department of Ophthalmology, New York Eye and Ear Infirmary of Mount Sinai, 310 East 14th Street, New York, NY 10003, USA

## Abstract

*Introduction*. We present a case of serpiginous choroidopathy (SC) with novel OCTA and en face OCT reflectance findings which help identify subclinical disease progression. *Case Presentation*. En face OCT reflectance images demonstrated outer retinal tubules (ORT) at the serpiginous lesion margins of affected and unaffected retina on multimodal imaging. OCTA findings demonstrate variable dropout of choriocapillaris in “normal” retina beyond lesion borders which was not visible on standard imaging and which demonstrated a clear transition zone beyond the ORT. *Discussion*. This is the first report of choriocapillaris atrophy identified on OCTA not identified on traditional multimodal imaging in serpiginous choroidopathy. Damage to vasculature only visible with OCTA may help characterize the distribution of inflammation, aiding in monitoring of suppression not illustrated by traditional imaging and which may threaten the central macula. ORT in SC suggest death and reorganization of outer segments from dysfunction of the choriocapillaris and RPE, as well as serve to demarcate the area of chronic or old inflammation, supporting the hypothesis that the choriocapillaris is the primary site of inflammation in SC. Based on these findings, we recommend OCTA on all patients with serpiginous choroidopathy to monitor underlying state of inflammation and help determine immunosuppressive threshold.

## 1. Introduction

The pathogenesis of SC is poorly understood, with clinical and histologic studies suggesting autoimmune, infectious, vasculopathic, and retinal degenerative etiologies [1, 2]. Recent advances in retinal imaging have ushered in a new era of new diagnostics in posterior uveitic diseases. Given the unclear cause from clinical and laboratory studies, much attention has shifted towards these new modalities. A patient presented with serpiginous choroidopathy (SC) and underwent OCTA and en face OCT reflectance imaging. We report novel OCTA and en face OCT reflectance imaging findings of outer retinal tubules (ORT) in SC.

## 2. Case Report

A 37-year-old female with no past medical or ocular history presented with 2 weeks of redness, pain, and photophobia of her right eye (OD). She was diagnosed with acute anterior uveitis by an outside provider and started on topical difluprednate four times daily (QID) and cyclopentolate once daily (QD) OD. Upon presentation to our facility, she reported slight improvement in her symptoms 2 weeks into treatment.

**Figure 1:**
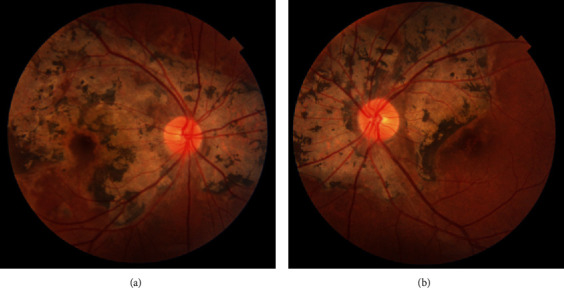
Color fundus photo of the right eye (a) and left eye (b).

On initial examination, visual acuity was 20/20 in her right and 20/20 in her left eye (OS) with unremarkable pupillary exam and normal intraocular pressure. Anterior segment examination of the right eye (OD) revealed 2 areas of anterior corneal stromal scarring and 0.5+ anterior chamber cell without flare. One area of anterior stromal scarring and trace cell without flare was seen OS. Fundoscopy revealed 0.5+ vitreous cell without haze in both eyes (OU); healthy appearing optic nerves OU; and extensive, serpentine peripapillary chorioretinal scarring OU with extension into the macula (Figures 1(a) and 1(b)). There were no hemorrhages or vascular sheathing, and the peripheral retina was unremarkable. Fluorescein angiography (FA) was performed, which demonstrated hyperfluorescence at the lesion margins visible on gross funduscopy. Fundus autofluorescence (FAF) demonstrated hyperautofluoresence at lesion margins and hypoautofluorescence in areas of atrophic retina. Pertinent laboratory workup included negative QuantiFERON-TB Gold, fluorescent treponemal antibody (FTA), and Lyme IgM and IgG. A diagnosis of serpiginous choroidopathy was made, and the patient started on oral prednisone 60 mg daily, with a taper of the difluprednate OD by one drop per week.

**Figure 2:**
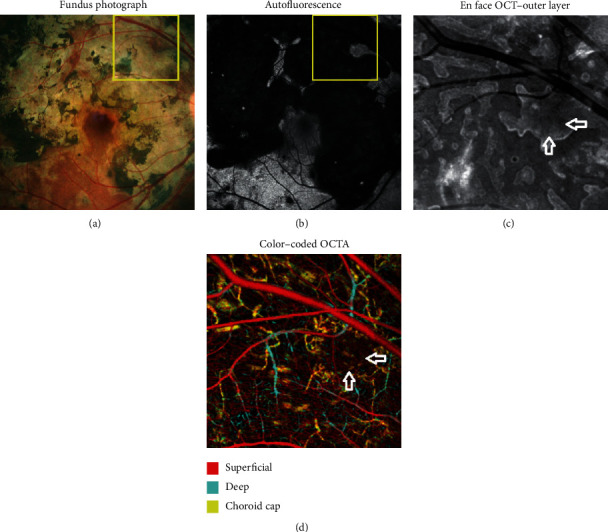
Composite images of the right eye: color fundus photo (a); fundus autofluorescence (b); magnified superficial en face OCT reflectance slab demonstrating ORT margins (c); and magnified color-coded OCTA of superficial, deep, and choriocapillaris vessels showing dropout beyond normal-appearing retina (d). White arrows demonstrate the corresponding area which is intact on fundus photo (a) and autofluorescence (b), lies within the border of the outer retinal tubules seen on en face imaging (c), but demonstrates patchy dropout of choriocapillaris identical to adjacent areas. Of note, there is increased visibility of choriocapillaris at the legion margins, seen as yellow-coded vessels and analogous to window defect.

The patient rapidly improved, and immunomodulatory therapy was initiated with a standard steroid taper. At one-month follow-up, en face OCT reflectance and OCTA imaging were obtained, which demonstrated severe atrophy with outer retinal tubules and patchy dropout of choriocapillaris in areas of otherwise normal-appearing retina, respectively (Figure 2). Over the next 2 months, the prednisone was slowly tapered and she continued mycophenolate mofetil 1 gram twice daily without recurrence of intraocular inflammation or activation of disease at the lesion margins. Repeat SD-OCT and OCTA imaging remained stable.

## 3. Discussion

We present a case of serpiginous choroidopathy (SC) imaged with OCTA and en face OCT reflectance imaging. Images were obtained and processed based upon previously described methodologies in the literature [3, 4].

OCTA imaging showed dropout of the choriocapillaris at lesion margins and discontinuously in adjacent areas centrally which appeared normal on traditional imaging such as FA (Figure 2(d)). Prior to obtaining OCTA, these areas closer to the fovea were presumed to be unaffected by SC based upon FA and examination. Fortunately, with high-dose oral steroids and immunomodulatory therapy, these subclinical areas remained stable and the patient did not experience further vision loss. Additionally, there was markedly increased visibility of the choriocapillaris between the outer retinal tubule and lesion borders, likely indicating some degree of reconstitution of choriocapillaris in affected retina and RPE atrophy improving visibility of underlying choroidal vasculature. This is consistent with prior reports [5].

OCT angiography allows a noninvasive assessment of choroidal and deep vasculature sometimes not evident on fluorescein angiography (FA). Recently, several small case series of OCTA in SC have suggested extensive inflammation in deep retinal vessels, RPE, and choriocapillaris. Desai et al. reported a series of 6 eyes of 3 patients with active and inactive SC. In this series, active patients demonstrated absence of choriocapillaris with thickening of overlying RPE and outer retinal layers on OCTA in areas of atrophic serpiginous lesions [5]. Inactive patients demonstrated partial reappearance of choriocapillaris at lesion margins with absence of outer retina, RPE, and choriocapillaris at inactive geographic regions [5]. Another case series of active and inactive SC lesions demonstrated disproportionate involvement of choriocapillaris compared to overlying RPE and deep retinal vasculature, suggesting that the choriocapillaris may be the primary pathologic site in SC [6]. In our patient, dropout of deep retinal vasculature was seen beyond lesion borders identified on photography and fluorescein angiography, indicating subclinical inflammation in areas not previously identifiable.

**Figure 3:**
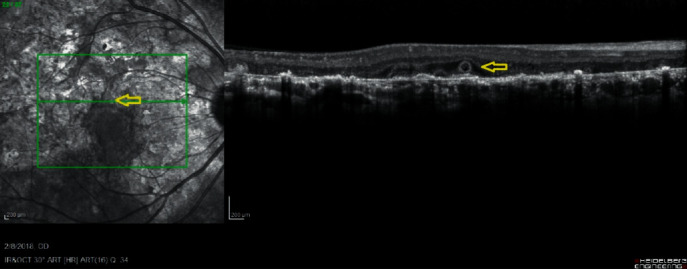
SD-OCT single-line raster of the right eye demonstrating superior parafoveal outer retinal tubule (yellow arrow).

We analyzed en face OCT reflectance image slabs, as well as cross-sectional OCT images. En face OCT reflectance imaging demonstrated outer retinal tubules at the quiescent serpiginous margins just adjacent to those areas of atrophic choriocapillaris on OCT which were normal-appearing on funduscopy and FA (Figure 2(c)). Cross-sectional OCT imaging confirmed the presence of ORT in areas of atrophic retina (Figure 3). To the best of our knowledge, only two reports of ORT in serpiginous choroidopathy exist [7, 8]. Our patient demonstrated outer retinal tubules adjacent to the serpiginous lesion borders between affected and nonaffected retina and choroid, but not within the atrophic areas themselves. Absence of ORT in areas of subclinical inflammation identified on OCTA indicates less chronic or severe inflammation in those areas, which would likely manifest without proper treatment. This may ultimately indicate inability of FA and OCT alone in identifying extent of risk in these patients and level of immunosuppression needed to preserve vision.

On SD-OCT imaging, outer retinal tubules appear as circular areas of hyporeflectivity with a ring of hyperreflectivity contained within the outer nuclear layer. Prior investigations of outer retinal tubules have centered around age-related macular degeneration (AMD) and spectral domain OCT (SD-OCT) but never reported in SC. Of the 69 eyes with outer retinal tubules identified in one study, 81% were attributable to AMD [9]. Other conditions observed with ORT included central serous chorioretinopathy, choroideremia, multifocal choroiditis with panuveitis, and Bietti crystalline dystrophy [9]. This finding is hypothesized to be secondary to reorganization of cone outer segments after loss of tight junction integrity. Histopathologic examinations of patients with outer retinal tubules have demonstrated ELM and inner segment mitochondria comprising the hyperreflective portion of the outer retinal tubules, with the luminal wall containing degenerated cone photoreceptors and RPE cells within the lumen [10, 11]. Given previously reported imaging and pathologic data of outer retinal tubules, this may indicate a shared anatomic pathway for SC and noninflammatory retinal degenerations. Regardless of pathogenesis, this case demonstrates chronic inflammation at serpiginous borders as reflected by ORT and OCTA transition zones, and more importantly, those areas of relative deep retinal and choriocapillaris loss on OCTA occur beyond those lesion borders.

## 4. Conclusion

Severe choriocapillaris inflammation may be the cause of outer retinal atrophy in serpiginous choroidopathy, with the presence of ORT signifying reorganization of photoreceptors localizing inflammation to this area. Current multimodal imaging without use of OCTA is widely accepted for the diagnosis and monitoring of SC. In this report, we describe a patient with OCTA findings of choriocapillaris atrophy demonstrating subclinical disease beyond serpiginous lesion borders visible on standard imaging. Loss of choriocapillaris integrity in areas outside of the serpiginous borders suggests subclinical activity which, if followed with OCTA, may aid in earlier identification of progression or more targeted timing for tapering of immunomodulatory therapy if stable. This suggests that OCTA may be more sensitive than SD-OCT and FA in identifying activity in SC and should be more widely adopted in the long-term management of these patients.
